# The Innovation of Entrepreneurship Education for Intangible Cultural Heritage Inheritance From the Perspective of Entrepreneurial Psychology

**DOI:** 10.3389/fpsyg.2022.721219

**Published:** 2022-03-31

**Authors:** Jie Zhou, Ji Qi, Xuefeng Shi

**Affiliations:** ^1^College of Liberal Arts, Shantou University, Shantou, China; ^2^College of Cultural Industries Management, Communication University of China, Beijing, China; ^3^Medical School Laboratory, The University of Chicago, Chicago, IL, United States

**Keywords:** entrepreneurial psychology, intangible cultural heritage, protection and inheritance, psychology perspective, small and micro enterprise entrepreneurship

## Abstract

The purpose is to help college students start their own businesses and protect and develop China’s intangible cultural heritage. The entrepreneurship of college students in the field of intangible culture is studied from the perspective of entrepreneurial psychology. First, the related characteristics, main content, and research status of college entrepreneurship education are described in detail. Entrepreneurial psychology is divided into entrepreneurial cognition, entrepreneurial emotion and entrepreneurial will. Then, the concept and development status of intangible cultural heritage are briefly summarized to illustrate the importance of inheriting and developing intangible cultural heritage. A questionnaire is designed based on intangible cultural heritage development mode to interview local college students randomly. Finally, the results are analyzed by descriptive statistics. The results show that massive students do not understand the entrepreneurial policy, and only 16% of them know it very well. More than 30% of the students think that entrepreneurship will improve personal responsibility. Nearly 50% of the students believe that entrepreneurship can increase their life experience. Almost 50% of the students have the psychological quality of persistence and courage in entrepreneurship. The final results show that there are great differences in entrepreneurial cognition among students of different grades. Regarding entrepreneurial emotion, there are obvious differences among students of different genders, disciplines, and grades. Regarding the entrepreneurial will, there are only differences among students with different grades. In entrepreneurship education, different teaching methods should be adopted for different ages to comprehensively improve the effect of entrepreneurship education for students of all grades and help them obtain good entrepreneurship results. This exploration provides technical support for the inheritance of material and cultural inheritance and contributes to the entrepreneurship education of college students.

## Introduction

The educational inheritance of intangible cultural heritage is an important measure to improve the quality of educational spiritual civilization. Therefore, the inheritance of campus intangible cultural heritage is an urgent national project. The key to campus inheritance lies in students, who are the protectors and inheritors of intangible cultural heritage. In cultivating high-quality, innovative, and application-oriented visual communication design talents, high quality is the foundation, the innovative ability is the core, and application oriented is the goal (Song et al., 2019). There are three ways for colleges to protect and inherit intangible cultural heritage. The first is to build college students’ deep recognition of local culture. The second is to establish a benign interaction mode between schools and non-governmental (communities). The third is to explore the industrialization path of intangible cultural heritage in the in-depth cooperation between schools and enterprises (Sun et al., 2021). Hence, colleges should strengthen the inheritance education of intangible cultural heritage in college students’ innovation and entrepreneurship education. Meanwhile, the inheritance of intangible cultural heritage also increases the entrepreneurial mode of college graduates and improves the employment rate (Ying and Wang, 2021). The educational work of colleges in the inheritance of intangible cultural heritage is not prominent, so more research is needed to provide more references.

Tan et al. (2020) pointed out an interactive relationship between intangible cultural heritage and entrepreneurship education for art college students, which was created in inheritance and innovated in protection. Therefore, the entrepreneurship education of art college students should focus on the protection and development of intangible cultural heritage. Besides, according to the characteristics of art colleges, it should also promote the construction of the entrepreneurship education system and entrepreneurship training mechanism from the four aspects of objectives, courses, practice, and teachers (Tan et al., 2020). Dan and Wen (2021) pointed out that a nation in a long history accumulated intangible cultural heritage. It is the precious wealth of the Chinese nation and a vital resource and spiritual guidance in building socialism. Due to economic globalization and urbanization acceleration, China’s mainstream culture is gradually replaced by foreign culture. Many intangible cultural heritages face the threat of forgetting, damage, and disappearance. As an essential base for cultivating high-quality talents, colleges should undertake the protection and inheritance of intangible cultural heritage. Moreover, intangible cultural heritage is rich in cultural resources, which is important for colleges to carry out education. Therefore, colleges should reasonably apply intangible cultural heritage to teaching according to the current situation to improve students’ humanistic quality and promote higher education development (Dan and Wen, 2021). Lambert and Rennie (2021) pointed out that only relying on economic growth could not improve the country’s influence and international status. It also needs some cultural outputs to make more international friends feel the charm of China’s traditional culture to realize the real national rejuvenation. China has a history of 5,000 years and countless excellent traditional cultures, including lots of intangible cultural heritages. Many have been lost in the long river of years because of their unique form. As China pays increasing attention to intangible cultural heritage, how to protect, inherit, carry forward, and innovate the intangible cultural heritage has become the most critical problem at present. In fact, intangible cultural heritage itself has no fixed expression forms and has different inheritance and development modes in different times. At present, the development of intangible cultural heritage in China is mainly reflected in theoretical publicity but lacks practical and systematic research (Lambert and Rennie, 2021).

Given the abovementioned analysis, entrepreneurship education of intangible cultural heritage inheritance is one of the crucial research directions at present. Therefore, this exploration takes the impact of innovative education of intangible cultural heritage inheritance on college students’ entrepreneurship as the starting point, divides entrepreneurial psychological quality into specific categories, and investigates college students’ personal quality and entrepreneurial ability through the development mode of intangible cultural heritage and college students’ cognition of intangible cultural heritage. By designing a questionnaire, college students’ entrepreneurial psychology is studied from three aspects: entrepreneurial cognition, entrepreneurial emotion, and entrepreneurial will. The existing problems are put forward, and the hidden value and far-reaching significance behind the intangible cultural heritage are deeply analyzed. This exploration provides technical support for the inheritance of intangible cultural heritage and provides an important guarantee for college students’ entrepreneurship.

### Entrepreneurship Education of Intangible Cultural Heritage Inheritance

#### Entrepreneurship Education Theory in Colleges

Higher education shoulders the crucial task of training qualified builders and reliable successors of socialist cause with all-round development of morality, intelligence, sports, and beauty (Karlsson, 2021). College graduates have experienced systematic higher education, and they are professionals with specific professional knowledge and skills. They are the new force and successors of socialist construction. Strengthening college students’ innovation and entrepreneurship education, cultivating their innovative spirit and entrepreneurial consciousness, cultivating positive and healthy innovation and entrepreneurship concepts, and strengthening the construction of ideological and political causes are the inevitable requirement of the smooth realization of the talent training plan of higher education (Huang, 2021). Besides, they are also crucial responsibilities of colleges’ current ideological and political education. Innovation and entrepreneurship education is a teaching concept and model that adapts to the needs of economic society and national development strategy. It is of great practical and strategic significance to promote innovation and entrepreneurship education in colleges to deepen the reform of education and teaching, promote the effective development of ideological and political education, and improve the quality of talent training (Joshua and Bendickson, 2020). Innovation and entrepreneurship education should be deeply into all students and reflected in the whole process of talent training.

Professional education is taken as the basis. Changing and renewing educational concepts are taken as guidance, and improving students’ sense of social responsibility, innovative spirit, entrepreneurial spirit, and entrepreneurial ability is taken as the core (Henche and Salvaj, 2017). The talent training mode and curriculum system are reformed. The innovation of entrepreneurship education and ideological and political education in colleges is promoted. The quality of talent training is constantly improved. Achieving full and high-quality employment and initiative entrepreneurship is the ultimate embodiment of college students’ personal and social values. It is also the need for college students’ all-round development (Pokidko, 2021). Combining online education development with college students’ innovation and entrepreneurship education and studying its effectiveness have become a crucial part of higher education in the new situation. Innovation and entrepreneurship education adheres to the employment needs of entrepreneurial students and solves the ideological confusion and psychological pressure of college students in employment and entrepreneurship. It helps students strengthen the primary consciousness and cultivates and improves the innovative spirit, entrepreneurial quality, and entrepreneurial spirit of college students to make them calmly face the challenges of reality and realize their self-worth (Chhabra et al., 2003).

#### Research Status and Main Theoretical Contents of Entrepreneurship Education

The innovation and entrepreneurship curriculum and the evaluation of education effect are based on innovation and entrepreneurship education content. The ability requirements of innovation and entrepreneurship for college students determine the content and objectives of innovation and entrepreneurship education (Lambert and Rennie, 2021). Meanwhile, they also determine the talent training orientation and development mode, the construction of discipline system, the development of teaching resources, and the teaching methods and training mode (Azzurra, 2020). Specifically, innovation and entrepreneurship education mainly includes the following aspects.

(1) Consciousness of innovation and entrepreneurship

The consciousness of innovation and entrepreneurship is a guiding ideology for entrepreneurs in the practice of innovation and entrepreneurship, which can stimulate and promote the practice of innovation and entrepreneurship. It is not born of innovation and entrepreneurship practitioners, but is produced by innovation and entrepreneurship practitioners’ scientific analysis, generalization, refinement, and sublimation of the reality, objective environment, and their own conditions (Racine, 2021).

(2) The spirit of innovation and entrepreneurship

College students’ innovation and entrepreneurship is an idea that runs through classroom teaching and extracurricular activities in colleges. It cultivates students’ innovation consciousness, creative spirit and entrepreneurial ability, and makes students boldly go to the society and start their own businesses after graduation. It mainly includes self-confidence, enterprising spirit, and sense of responsibility, emphasizing the independence of life and the courage and confidence to face life independently and meet challenges. The thinking set and framework of unconventional exploration and meeting challenges, with a wide range of humanistic care, fully shows individuals’ moral and legal responsibility to the society, the country and others, and consciously fulfill this responsibility (Shekhar and Huang, 2021).

(3) The ability of innovation and entrepreneurship

The ability of innovation and entrepreneurship generally refers to the basic knowledge of innovation and entrepreneurship, which is accumulated through learning and relevant experience. After systematic and scientific processing, new concepts, new knowledge, and methods are generated. The ability of consciousness and practice is the embodiment of their ability content. They are inseparable, which are reflected in college graduates’ practice, innovation, and entrepreneurship. Innovation capability is a concept and a unity of many factors (Yuan, 2020).

(4) Innovation and entrepreneurship practice

As a crucial part of innovation and entrepreneurship education, innovation and entrepreneurship practice tests and promotes the innovation and entrepreneurship theory. There are various innovation and entrepreneurship practice activities. Through these activities, innovation entrepreneurs can combine the theoretical knowledge they have learned with practice to test the understanding degree of knowledge and existing problems in practice (Ahmad et al., 2019).

#### Inheritance Theory of Intangible Cultural Heritage

**FIGURE 1:**
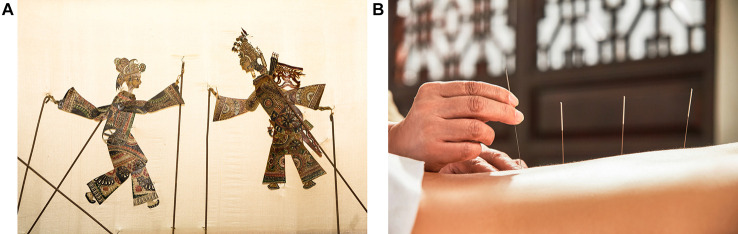
The intangible cultural heritage [**(A)** shadow puppets and **(B)** acupuncture]. Royalty free images reproduced with permission from Shutterstock.

Intangible cultural heritage is the precious experience that people’s ancestors summed up through hard work and sweat in the long river of historical development. It includes cultural knowledge and life art, some traditional and unique skills, which are valuable resources worthy of inheritance and development (Carrillo and Lang, 2021). Figure 1 shows the intangible cultural heritage.

**FIGURE 2:**
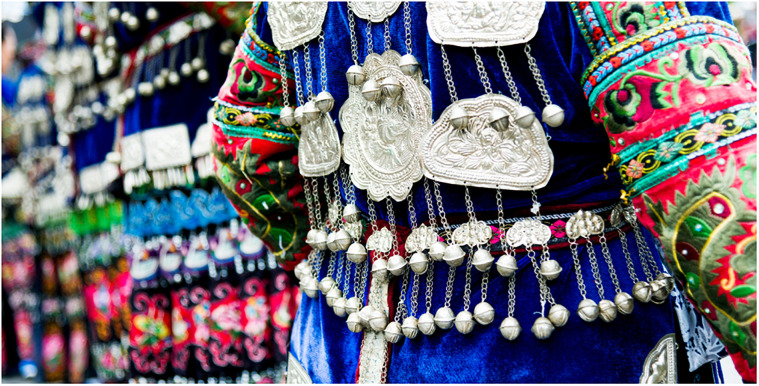
Teaching of Miao costume making. Royalty free images reproduced with permission from Shutterstock.

More than 47,000 shadow puppets and related cultural relics were donated by the famous collector Professor Zhao Shutong in November 2003. Based on this, the Shadow Puppet Art Museum of the Chinese Academy of Fine Arts is established, which is the first large-scale Shadow Puppet Art Museum in China. The collection of more than 45,000 shadow puppets comes from Shaanxi, Gansu, Shanxi, Hebei, Tangshan, Beijing, Henan, Hunan, Hubei, Sichuan, and other places from the Ming and Qing Dynasties to modern times. These shadow puppets have a long history, a wide variety, exquisite carving, and unique shape. Besides, there are 1,368 shadow puppet scripts, more than 400 shadow puppet bags and a set of performance musical instruments. In 2015, the shadow play art museum was merged into the Folk Art Museum of China Academy of Fine Arts (Deng, 2021). Acupuncture consists of “needle” and “Moxibustion,” which is one of the crucial components of Oriental Medicine. Its contents include acupuncture theory, acupoints, acupuncture technology, and related instruments. The process of its formation, application, and development has distinct Chinese national culture and regional characteristics. It is a valuable heritage based on Chinese national culture and scientific tradition (Liu, 2019). Intangible cultural heritage is a kind of Chinese traditional culture, a brilliant achievement of national culture and a concentrated embodiment of ancient civilization. As an ancient civilization with a history of 5,000 years, China must cherish and carry forward the existing intangible cultural heritage (Halder and Sarda, 2021). Nowadays, China has focused more on the intangible cultural heritage and strengthened the protection education of intangible cultural heritage (Guan, 2021). It is hoped that the successors for the socialist cause can inherit the intangible cultural heritage and innovate based on this. At present, the inheritance education of intangible cultural heritage mainly includes two aspects. (1) The inheritance of intangible cultural heritage can help students establish the correct concept and value system of art and beauty, and guide them to produce artistic resonance (Heredia, 2021). Meanwhile, most intangible cultural heritages contain the ancient people’s profound feelings of home and country and cultural cultivation, which can also help students cultivate patriotism and national righteousness, learn certain historical and cultural knowledge, expand their horizons, and cultivate noble ones’ sentiments (Lim, 2021). (2) The inheritance education of intangible cultural heritage is added to the college entrepreneurship education. It can exert a subtle cultural influence on college students and stimulate their entrepreneurial interest and willingness and help them open up a new entrepreneurial field. College students are about to enter the society and become successors of socialism, so they must fully realize intangible cultural heritage’s importance and far-reaching significance (Xu, 2021). The inheritance and protection of intangible cultural heritage may not get huge economic benefits in the short term. However, young college students must realize that economic interests are not the primary goal of life, and it is impossible to really feel the profound Chinese culture in a life driven by interests. The inheritance of intangible cultural heritage needs patience, perseverance, and calmness. If the intangible cultural heritage can be truly inherited and developed, its impact will be immeasurable (Henche and Salvaj, 2020). Figure 2 shows the on-site teaching of Miao costume production.

The Miao costume in Figure 2 has been listed in the national intangible cultural heritage list, and its inheritance depends on continuous production, teaching, and transmission from generation to generation (Guo, 2020). Table 1 presents the main inheritance methods of intangible cultural heritage.

**TABLE 1:** The main inheritance methods of intangible cultural heritage.

Inheritance methods	Concrete measures
Keep in the archives	1. The State Archives Bureau collects information and data of intangible cultural inheritance from the public; 2. Displaying them in museums; 3. Recording audio, video and other materials for intangible cultural heritage preservation.
Protection	1. Stabilizing and establishing the status of intangible cultural heritage; 2. Giving economic encouragement to the inheritors of intangible cultural heritage.
Propagation	1. Holding various kinds of intangible cultural heritage exhibitions; 2. Providing funds to help intangible cultural heritage advertising; 3. Providing support and material help for non-governmental intangible cultural heritage institutions.
Development	1. Carrying out project cooperation on intangible cultural heritage across fields and regions; 2. Carrying out international cooperation and exchanges.

Innovations in intangible cultural heritage are also crucial but they must be based on the knowledge already known. For young college students, the innovation of intangible cultural heritage is the concentrated embodiment of patriotism and nationalism and the expression of national confidence and cultural confidence. China has been influenced by western culture and western thinking for a long time, so the behavior of worshiping foreigners is very serious among college students. Students are prone to produce the psychology of distrust in the motherland culture (González, 2016). If things continue this way, college students gradually will fall into the cage of hedonism, not thinking enterprising, greedy to enjoy. Moreover, the ideology of college students seriously degenerates, their will is depressed, and they lack the planning of their own life goals. Thereby, intangible cultural heritage is of great significance to college students’ entrepreneurship education and ideological and political education (Moradi, 2021). The reason is that intangible cultural heritage contains the cultural heritage of China for 1,000 years, and is the precious spiritual wealth left by ancestors to future generations. An increasing number of people only focus on material enjoyment, ignore spiritual pursuit, and gradually become slaves of desire with the development and progress of society. College student is a young group about to enter society, while their minds have not yet mature, so they are easy to be tempted. In particular, they are more likely to lose will when facing social pressure. Intangible cultural heritage can help college students to cultivate firm will and belief, and cultivate their patriotism and national self-confidence (Yu and Ke, 2021). From the perspective of education, colleges must realize that the inheritance and innovation of intangible cultural heritage are of great significance to the education of college students. It can help college students cultivate noble sentiment and cultivation, carry forward traditional culture, and build up the national confidence of the students. More importantly, intangible cultural heritage is not only a theory, but also an art (Vander and Vanneste, 2015). Thereby, the integration of intangible cultural heritage requires college students to start from practice to cultivate their practical ability and promote the transition from theoretical study to social practice.

#### Research Methods of Entrepreneurship Education in the Inheritance of Intangible Cultural Heritage

Based on the abovementioned theories, it is believed that multiple influencing factors restrict the cultivation of college students’ entrepreneurial psychological quality, and it is gradually formed under the intervention of colleges and the government. Hence, the investigation and research on college students’ entrepreneurial psychological quality should start from three aspects: entrepreneurial cognition, entrepreneurial emotion, and entrepreneurial will (Miranda et al., 2020).

Entrepreneurial cognition refers to entrepreneurs’ cognition of relevant entrepreneurship support policies, entrepreneurship education in colleges, self-entrepreneurship purpose, entrepreneurship-related laws and regulations, and entrepreneurship ecological environment.

Entrepreneurial emotion refers to the inner driving factors of entrepreneurs, including personal responsibility, life experience, self-improvement, obtaining economic benefits, and enriching life experience.

Entrepreneurial will refers to the excellent qualities that entrepreneurs need to overcome difficulties in the process of entrepreneurship, including persistence, courage, effort, rationality, and self-discipline (Zhi, 2021).

A questionnaire about college students’ entrepreneurial psychological quality is designed based on entrepreneurial psychology by consulting relevant literature and materials. A random survey is conducted among local college students. After students are selected, they will be asked whether their schools carry out entrepreneurship education on intangible cultural heritage inheritance, and whether they have received such entrepreneurship education. The randomly surveyed students refer to those who have received entrepreneurship education for intangible cultural heritage inheritance in their school (Zhou et al., 2019). Moreover, the questionnaires are distributed and collected through the digital platform. First, the basic format of the questionnaire is obtained through the digital platform. Then, the designed questionnaire is distributed and recovered through the information exchange level to ensure the timing and efficiency of the questionnaire survey.

Participants: Overall, 400 local college students voluntarily participate in the survey. Questionnaires are distributed to them. In the survey process, the students’ psychological state and physical quality are ensured to be good, their understanding is normal, and their self-positioning is more accurate. Finally, 397 valid questionnaires are collected.

Survey methods: the questionnaires are sorted out, and the data are collected according to different genders, grades, and disciplines. Different dimensions of entrepreneurial psychological quality, such as entrepreneurial cognition, entrepreneurial emotion, and entrepreneurial will, are classified to perform descriptive statistics. Finally, the statistical data are qualitatively and quantitatively analyzed. The results are obtained, and the internal laws and reasons are analyzed.

Data processing methods: SPSS22.0 software is adopted to analyze the questionnaire data. The statistical data of students of different disciplines, genders, and grades are drawn according to the different dimensions of entrepreneurial psychological quality to intuitively conduct descriptive analysis. The *t*-test is also needed for different influencing factors. Whether the difference is obvious is judged according to the value of *p*, and the internal reasons are analyzed.

Content of the questionnaire: the content of the questionnaire mainly refers to the content and overall structure of the “questionnaire on college students’ entrepreneurial psychological quality” compiled by Han Lizheng, a scholar. Then, the questionnaire of college students’ entrepreneurial psychological quality of intangible cultural heritage is self-compiled. The main content includes three dimensions: entrepreneurial cognition, entrepreneurial emotion, and entrepreneurial will. Students’ personal basic information, such as age, gender, subject type, and grade, is also included. There are some innovations in the research contents and methods.

Evaluation criteria: the problems of entrepreneurial cognition in the questionnaire mainly include: entrepreneurship-related supporting policies, entrepreneurship education, entrepreneurial purpose, entrepreneurship-related laws and regulations, and entrepreneurship ecological environment. The score is divided into five grades, from 1 to 5. The higher the score is, the worse the cognitive level is. 1 is very understanding, while 5 is not understanding at all.

The problems of entrepreneurial emotion include personal responsibility, life experience, self-improvement, economic benefits obtaining, and rich life experience. There are five grades, from 1 to 5. The higher the score is, the lower the entrepreneurial emotion is. 1 represents that entrepreneurship can improve the sense of personal responsibility and others, while 5 means that entrepreneurship exerts no effect on themselves.

The problems of entrepreneurial will are persistence, courage, effort, rationality, and self-discipline. The score has five grades, from 1 to 5. The higher the score is, the weaker the will is. 1 is very strong, while 5 is not.

## Analysis of Research Results

### An Analysis of the Entrepreneurial Cognition of Intangible Cultural Heritage

**FIGURE 3:**
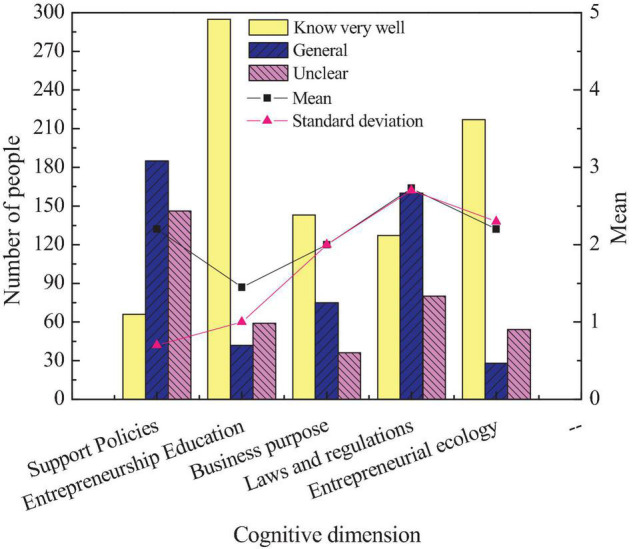
Students’ entrepreneurial cognition of intangible cultural heritage.

The first part of the questionnaire is the students’ cognition of entrepreneurship education on intangible cultural heritage inheritance. This survey can preliminarily understand students’ entrepreneurship and education level of intangible cultural heritage inheritance to understand the current work status of intangible cultural heritage inheritance education in colleges. Figure 3 reveals the survey results of college students’ cognition of entrepreneurship education on the inheritance of intangible culture.

**FIGURE 4:**
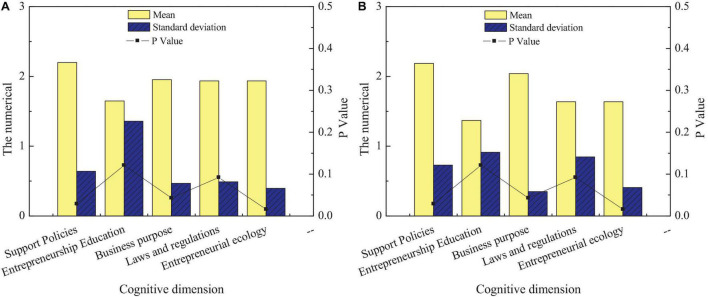
Differences in entrepreneurial cognition of intangible cultural heritage among students of different genders: **(A)** men and **(B)** women.

Figure 3 shows that many students’ understanding of the entrepreneurship policy of intangible cultural heritage inheritance is not ideal, and the proportion of students who do not understand is as high as about 40%. Next, students’ cognition of entrepreneurship education for intangible cultural heritage inheritance is relatively ideal, and more than 70% of students know it very well. It suggests that the school’s publicity of entrepreneurship education on intangible cultural heritage inheritance is relatively perfect. Finally, among the students’ cognition of the entrepreneurial purpose of intangible cultural heritage inheritance and relevant laws and regulations, the former is not ideal, and only about 35% of the students have a certain cognition of their entrepreneurial purpose. The latter is relatively ideal, and only about 20% of the students do not understand relevant laws and regulations. Generally, entrepreneurship education for intangible cultural heritage inheritance needs to be strengthened. The second research content of the questionnaire is to investigate the cognition of students of different genders on entrepreneurship education of intangible cultural heritage inheritance. Figure 4 shows the cognition of students of different genders on entrepreneurship education of intangible cultural heritage inheritance.

**FIGURE 5:**
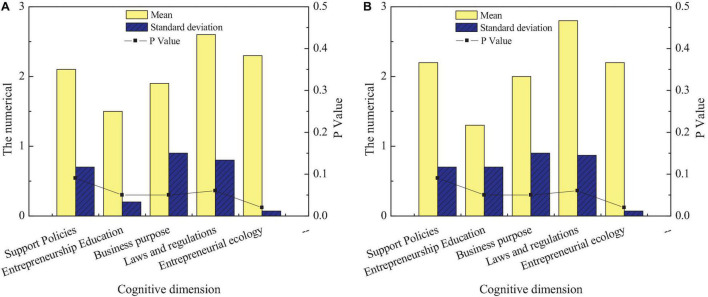
Comparison of students’ entrepreneurial cognition of intangible cultural heritage in different disciplines: **(A)** liberal arts and **(B)** science.

Figure 4 reveals that students of different genders have relatively small differences in their cognition of entrepreneurship education for intangible cultural heritage inheritance. First, for relevant entrepreneurship support policies, there are significant differences in the cognitive level of male and female students, and the cognitive level of female students is lower than that of male students. Next, there is no significant difference between male and female students in entrepreneurship education and relevant laws and regulations for the inheritance of intangible cultural heritage (*p* > 0.05). Finally, there is a significant difference between male and female students regarding entrepreneurial purpose and entrepreneurial environment of intangible cultural heritage inheritance (*p* < 0.05). Men are clearer about their entrepreneurial purpose than women, but women understanding of the entrepreneurial environment is clearer than men. The third content of this questionnaire is to investigate students’ cognition in different disciplines on entrepreneurship education of intangible cultural heritage inheritance. This survey can understand the degree of entrepreneurship education on intangible cultural heritage inheritance among different disciplines to improve the achievements of entrepreneurship education on intangible cultural heritage inheritance of the school from the perspective of disciplines. Figure 5 shows the comparison of entrepreneurial cognition of students from different disciplines on the inheritance of intangible cultural heritage.

**FIGURE 6:**
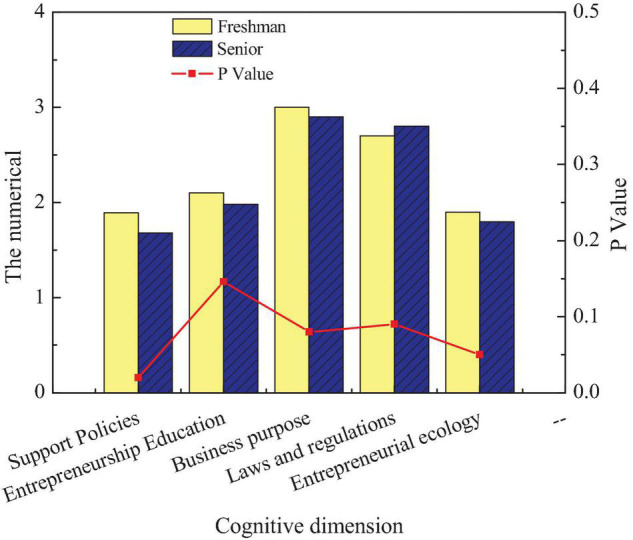
Comparison of entrepreneurial cognition among students of different grades.

Figure 5 reveals that except for the obvious cognition of entrepreneurship environment of intangible cultural heritage among different disciplines (*p* < 0.05), there are small differences in the cognition of students from different disciplines (*p* > 0.05). Among them, science students’ cognition of entrepreneurial environment is higher than liberal art students. The fourth content of the questionnaire is to investigate and study the entrepreneurial education cognition of intangible cultural heritage inheritance by students of different grades. Figure 6 displays the cognition of students of different grades on entrepreneurship education of intangible cultural heritage inheritance of the school.

Figure 6 reveals that students of different grades have apparent differences in their cognition of entrepreneurship-related support policies and entrepreneurial environment for intangible cultural heritage inheritance (*p* < 0.05). Compared with freshmen, senior students know more about supporting policies and entrepreneurial environment, but there is little difference between different grades in other aspects (*p* > 0.05).

### An Analysis of Students’ Entrepreneurial Emotion Toward Intangible Cultural Heritage

**FIGURE 7:**
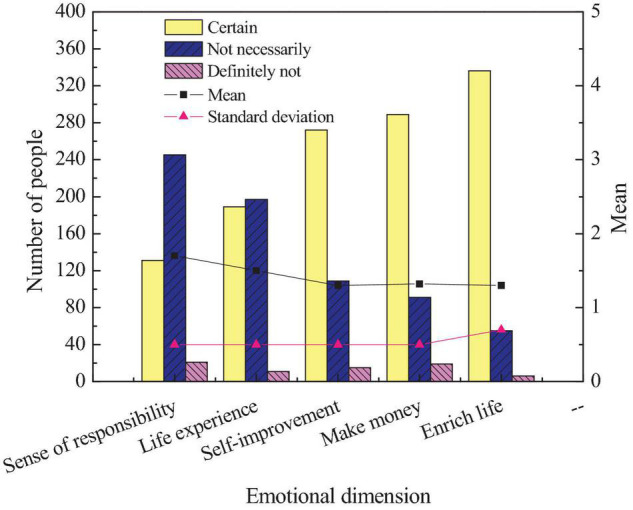
Comparison of students’ entrepreneurial emotion.

This exploration is mainly to study the entrepreneurship education of intangible cultural heritage inheritance through entrepreneurship psychology, so the research on students’ psychology is the leading research content. Thereby, the second content of the questionnaire is to research and investigate students’ entrepreneurial education emotion of intangible cultural heritage inheritance. Figure 7 shows the survey of students’ entrepreneurial emotions for intangible cultural heritage inheritance.

**FIGURE 8:**
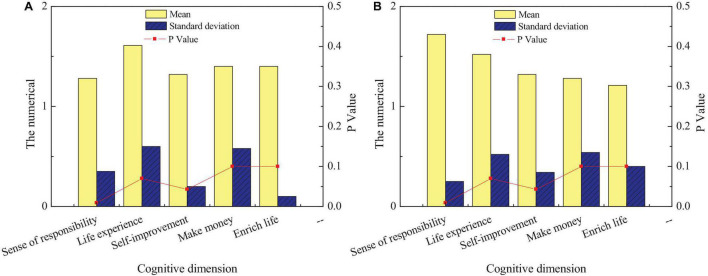
Comparison of entrepreneurial emotion among students of different genders: **(A)** men and **(B)** women.

Figure 7 reveals that, among the students’ entrepreneurial emotion for intangible cultural heritage inheritance, more than 30% of the students believe that entrepreneurship will improve their sense of personal responsibility. Nearly 50% of students believe that entrepreneurship can increase life experience. About 70% believe that entrepreneurship can improve themselves. More than 70% believe that entrepreneurship can bring economic benefits. More than 80% of students agree that entrepreneurship can enrich their lives. Given the abovementioned results, students’ entrepreneurial emotion for intangible cultural heritage inheritance tends to be positive as a whole. The entrepreneurial emotion of students of different genders on the inheritance of intangible cultural heritage is first studied. Figure 8 shows the survey results of entrepreneurial emotion of intangible cultural heritage inheritance of different students.

**FIGURE 9:**
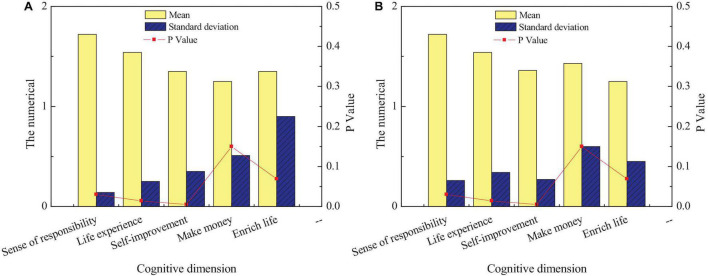
Comparison of entrepreneurial emotion among students of different disciplines: **(A)** liberal arts and **(B)** science.

Figure 8 suggests that there are obvious differences between men and women in terms of personal responsibility (*p* < 0.05); moreover, the score of men is lower than that of women, indicating that men have a stronger emotion to improve their personal responsibility in the entrepreneurial process of intangible cultural heritage inheritance than women; the difference between men and women is also obvious in terms of self-improvement. The average score of women is higher than that of men, indicating that men are more inclined to entrepreneurship than women; there is no significant difference between them in other aspects (*p* > 0.05). In the research on the entrepreneurial emotion of intangible cultural heritage inheritance, the comparison between different subjects is investigated. Figure 9 displays the emotional survey among different science students.

**FIGURE 10:**
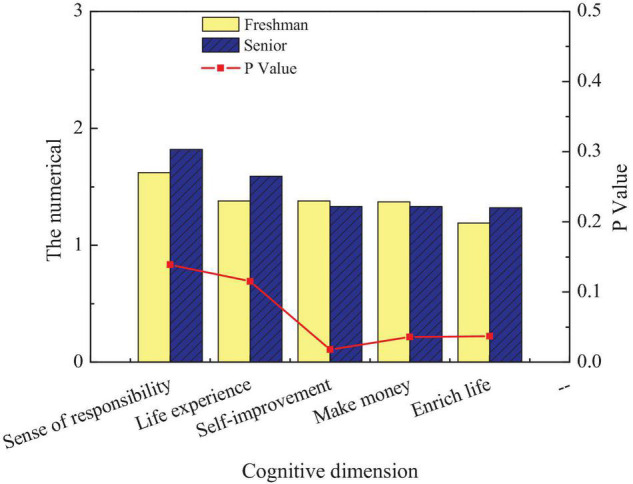
Comparison of freshmen and senior students’ entrepreneurial emotion.

Figure 9 shows significant differences among different disciplines in improving their sense of responsibility (*p* < 0.05). Science students have stronger feelings for improving their sense of responsibility than liberal arts students. Meanwhile, the scores of liberal arts students are higher than those of science students in life experience, with an obvious difference (*p* < 0.05). It suggests that science students are less satisfied with life experience than science students. In addition, liberal arts students also score higher than science students in terms of self-improvement, with a significant difference (*p* < 0.05). It indicates that science students focus more on self-improvement brought by entrepreneurship than liberal arts students. The entrepreneurial emotion of students in different grades for the inheritance of intangible cultural heritage is investigated. Figure 10 shows the comparison of entrepreneurial emotions of students of different ages on the inheritance of intangible cultural heritage.

Figure 10 reveals that freshmen and seniors have obvious differences in three aspects: self-improvement, obtaining economic benefits, and enriching life (*p* < 0.05). Among them, freshmen score higher than senior students in self-improvement and obtaining economic benefits, indicating that senior students focus more on self-improvement and economic benefits; the scores of freshmen in enriching life are lower than those of seniors. This phenomenon may be caused by the different entrepreneurial time between seniors and freshmen.

### Analysis of Students’ Entrepreneurial Will to Intangible Cultural Heritage

**FIGURE 11:**
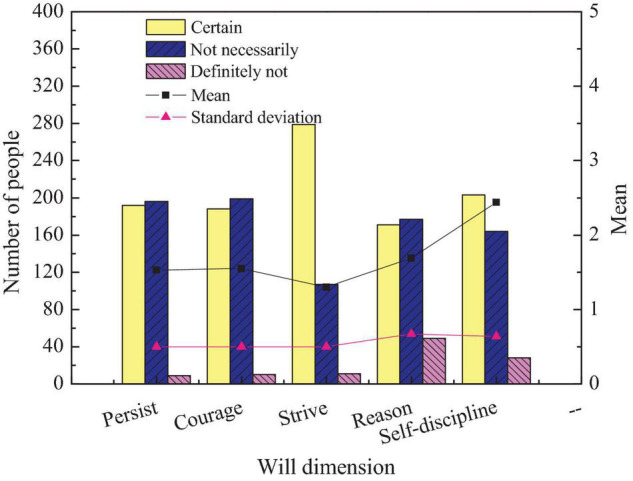
Comparison of students’ entrepreneurial will.

The third content of the questionnaire is to investigate the entrepreneurial will of students to inherit intangible cultural heritage. The impact of entrepreneurship education on students’ personal quality can be studied through this survey. Figure 11 shows the survey results of college students’ entrepreneurial will to inherit intangible cultural heritage in the entrepreneurial process.

**FIGURE 12:**
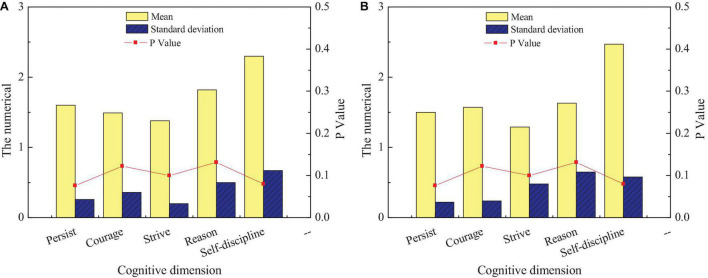
Comparison of entrepreneurial will of students of different genders: **(A)** men and **(B)** women.

Figure 11 suggests that nearly 50% of students have the psychological quality of persistence and courage to work hard in entrepreneurship. More than 70% of students believe that they put all their efforts into entrepreneurship. More than 40% of students believe that they need to be rational in entrepreneurship and cannot blame blindly. More than 50% of students believe that they need to maintain self-discipline in the entrepreneurship. The above data suggest that the entrepreneurial will of the vast majority of students tends to be positive. The entrepreneurial will of students of different genders is investigated. Figure 12 shows the comparison of entrepreneurial will of students of different genders.

**FIGURE 13:**
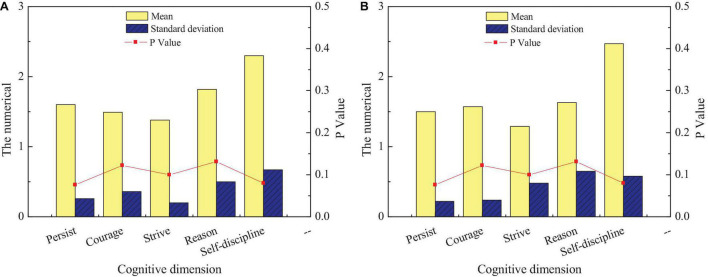
Comparison of entrepreneurial will among students of different disciplines: **(A)** liberal arts and **(B)** science.

Figure 12 shows that the scores of men and women in all dimensions of entrepreneurial will are basically the same, and there is no significant difference (*p* < 0.05), indicating that gender is not a factor affecting entrepreneurial will, and there is no significant difference between men and women entrepreneurial will. Overall, the score remains between 1 and 2, indicating that the psychological quality of entrepreneurial will is excellent. The entrepreneurial will of students in different disciplines to inherit intangible cultural heritage is studied. Figure 13 shows the comparison of entrepreneurial will among students of different disciplines.

Figure 13 displays that there is little difference between liberal art and science students in different dimensions of entrepreneurial will. Only in terms of self-discipline, the score of science students is higher than that of liberal arts students, and the difference is obvious (*p* < 0.05). It is stated that science students are more self-disciplined than science students in entrepreneurship, but there is no significant difference in other aspects (*p* > 0.05).

**FIGURE 14:**
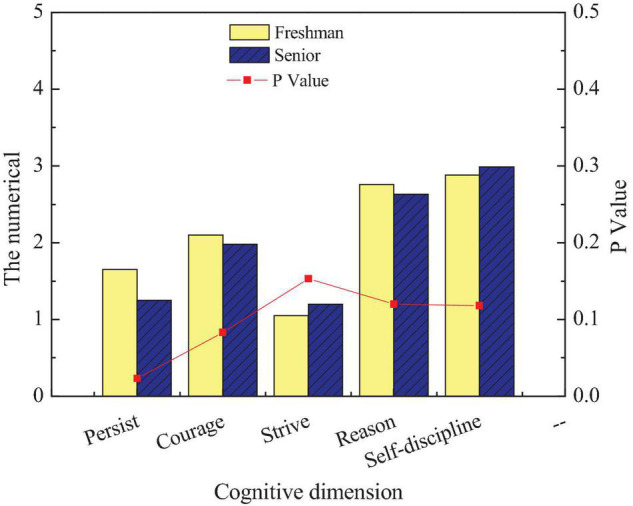
The comparison of entrepreneurial will among students of different grades.

The entrepreneurial will of students in different grades to inherit intangible cultural heritage is studied. Figure 14 shows the comparison of the entrepreneurial will of students in different grades.

Figure 14 shows significant differences in the scores of persistence among students of different grades (*p* < 0.05). The score of freshmen is higher than that of senior students, indicating that their persistence is not as good as that of senior students. The possible reason is that after 4 years of entrepreneurship education and environmental edification, the persistence characteristics of college students have been honed and improved.

## Discussion

This exploration is to study the innovation of entrepreneurship education in the inheritance of intangible cultural heritage from entrepreneurial psychology. The results show that the publicity work of contemporary entrepreneurship education is outstanding, and 70% of students hold that they know very well about entrepreneurship education of intangible cultural heritage inheritance. However, students of different genders have different perceptions of various conditions for entrepreneurship. The school’s entrepreneurial support for students of different genders is the same, but the performance of different genders is very different, which shows that students of different genders hold different attitudes and have a different enthusiasm for entrepreneurial education of intangible cultural inheritance. Meanwhile, men have a stronger awareness of entrepreneurial responsibility than women. Moreover, under the same conditions of entrepreneurship education in schools, there are significant differences in students’ cognition of entrepreneurship education environment in different disciplines, especially between science students and liberal art students. Moreover, there are significant differences in students’ cognition of entrepreneurial responsibility between the two disciplines. Finally, the higher the grade is, the higher the students’ cognition of all aspects of entrepreneurship education of intangible cultural heritage inheritance is; the higher the grade is, the better the students’ performance in entrepreneurial enthusiasm and entrepreneurial responsibility is. The abovementioned results show that there are still multiple deficiencies in the education of inheriting material and cultural heritage. First, schools should adopt different degrees of entrepreneurship education according to different genders, disciplines, and grades to strengthen the comprehensive development of entrepreneurship education. Then, schools should strengthen the education of entrepreneurship conditions with low students’ cognition, especially entrepreneurship policies, which need to be comprehensively strengthened. Finally, strengthening students’ entrepreneurship education can improve the school’s education quality and comprehensive quality, create more entrepreneurial ways for students, and improve their entrepreneurial success rate. The gender gap, age gap, and subject gap of students’ entrepreneurship are analyzed in detail. The 4-year teaching of entrepreneurship theory course for college students has stimulated and promoted the practice of innovation and entrepreneurship, cultivated students’ innovative consciousness, spirit and ability, and made the entrepreneurial will of the vast majority of students tend to be positive. Moreover, the characteristics of education make the effect of innovative theory course for science and engineering students better than liberal arts students. It improves students’ self-confidence, enterprising spirit and sense of responsibility, and enhances their courage and confidence to face life and meet challenges independently. It provides a non-traditional mode of thinking and framework for exploring and meeting challenges, as well as extensive humanistic care. Moreover, it fully shows the individual’s moral and legal responsibilities to the society, the state, and others, so that the theoretical knowledge learned in innovation and entrepreneurship course can be combined with practice, test the understanding of knowledge and the problems existing in practice, and promote students to consciously fulfill this responsibility. Compared with Watson and Mcgowan (2019), the fields involved here are more in-depth and the methods used are more advanced, which provides more educational ideas for entrepreneurship education. Compared with the research results of Chen (2021), the difference comparison in entrepreneurial awareness and ability between students of different genders and different disciplines have been added.

## Conclusion

Entrepreneurial psychology is adopted to study the entrepreneurship education of college students under the background of college and society advocating college students’ innovation and entrepreneurship. The entrepreneurial psychology of college students is divided into three aspects: entrepreneurial cognition, entrepreneurial emotion, and entrepreneurial will. A questionnaire is designed and a descriptive statistical analysis of entrepreneurial psychology is conducted according to different genders, disciplines, and grades. The results show great differences in entrepreneurial cognition among students of different grades. Regarding entrepreneurial emotion, there are obvious differences among students of different genders, disciplines, and grades; there are only differences in the entrepreneurial will among students with different grades. The research deficiencies are as follows. First, the publicity of intangible cultural heritage is not perfect in the society, so there are not many colleges carrying out intangible cultural heritage inheritance and entrepreneurship education, and it is necessary to find such schools specifically. Then, there are many intangible cultural heritage projects, and different majors and schools have different emphases on entrepreneurship education, so more detailed research is needed. Based on the above deficiencies, future research will focus on strengthening the analysis of the influencing factors of intangible cultural heritage entrepreneurship education, increasing the innovation of information acquisition methods, and deeply exploring intangible cultural heritage itself. Under the influence of traditional education methods, college education was used to be the main platform for the world with academic practice, and it cultivated massive high-knowledge and high-quality talents for the country and society. However, with the development of society, a single academic talent training is no longer fully suitable for the needs of social development. Throughout modern society, the development of all walks of life not only needs professional academic talents who lead high technology, high skills, and high knowledge, but also needs technical talents who combine theory and practice. Therefore, carrying out the innovation and entrepreneurship education of intangible cultural heritage inheritance in colleges means that China’s college education is a further deepening reform. The main purpose of its reform is to further integrate higher education with social development. It not only promotes the inheritance of intangible cultural heritage but also stimulates the development of college students’ employment and social innovation.

## Data Availability Statement

The raw data supporting the conclusions of this article will be made available by the authors, without undue reservation.

## Ethics Statement

The studies involving human participants were reviewed and approved by the Shantou University Ethics Committee. The patients/participants provided their written informed consent to participate in this study. Written informed consent was obtained from the individual(s) for the publication of any potentially identifiable images or data included in this article.

## Author Contributions

All authors listed have made a substantial, direct, and intellectual contribution to the work, and approved it for publication.

## Conflict of Interest

The authors declare that the research was conducted in the absence of any commercial or financial relationships that could be construed as a potential conflict of interest.

## Publisher’s Note

All claims expressed in this article are solely those of the authors and do not necessarily represent those of their affiliated organizations, or those of the publisher, the editors and the reviewers. Any product that may be evaluated in this article, or claim that may be made by its manufacturer, is not guaranteed or endorsed by the publisher.
